# Loss to Follow-Up from HIV Screening to ART Initiation in Rural China

**DOI:** 10.1371/journal.pone.0164346

**Published:** 2016-10-21

**Authors:** Diane Gu, Yurong Mao, Zhenzhu Tang, Julio Montaner, Zhiyong Shen, Qiuying Zhu, Roger Detels, Xia Jin, Ran Xiong, Juan Xu, Walter Ling, Lynda Erinoff, Robert Lindblad, David Liu, Paul Van Veldhuisen, Albert Hasson, Zunyou Wu

**Affiliations:** 1 The National Center for AIDS/STD Control and Prevention, Chinese Center for Disease Control and Prevention, Beijing 102206, China; 2 Guangxi Center of Disease Control and Prevention, Nanning, Guangxi, China; 3 BC Center for Excellence in HIV/AIDS, University of British Columbia, Vancouver, BC, Canada; 4 Department of Epidemiology, UCLA Fielding School of Public Health, Los Angeles, CA, United States of America; 5 Integrated Substance Abuse Programs, David Geffen School of Medicine at UCLA, Los Angeles, CA, United States of America; 6 National Institute on Drug Abuse, National Institutes of Health, Bethesda, MD, USA; 7 The EMMES Corporation, Rockville, MD, United States of America; Médecins Sans Frontières (MSF), INDIA

## Abstract

**Background:**

Patients who are newly screened HIV positive by EIA are lost to follow-up due to complicated HIV testing procedures. Because this is the first step in care, it affects the entire continuum of care. This is a particular concern in rural China.

**Objective(s):**

To assess the routine HIV testing completeness and treatment initiation rates at 18 county-level general hospitals in rural Guangxi.

**Methods:**

We reviewed original hospital HIV screening records. Investigators also engaged with hospital leaders and key personnel involved in HIV prevention activities to characterize in detail the routine care practices in place at each county.

**Results:**

699 newly screened HIV-positive patients between January 1 and June 30, 2013 across the 18 hospitals were included in the study. The proportion of confirmatory testing across the 18 hospitals ranged from 14% to 87% (mean of 43%), and the proportion of newly diagnosed individuals successfully initiated antiretroviral treatment across the hospitals ranged from 3% to 67% (mean of 23%). The average interval within hospitals for individuals to receive the Western Blot (WB) and CD4 test results from HIV positive screening (i.e. achieving testing completion) ranged from 14–116 days (mean of 41.7 days) across the hospitals. The shortest interval from receiving a positive EIA screening test result to receiving WB and CD4 testing and counseling was 0 day and the longest was 260 days.

**Conclusion:**

The proportion of patients newly screened HIV positive that completed the necessary testing procedures for HIV confirmation and received ART was very low. Interventions are urgently needed to remove barriers so that HIV patients can have timely access to HIV/AIDS treatment and care in rural China.

## Introduction

Studies worldwide have confirmed the life-saving benefits of the early initiation of antiretroviral therapy (ART) for people living with HIV (PLHIV) [1–4]. Most HIV-infected patients are able to achieve viral suppression once they are successfully engaged in ART [5]. Over the past decade, China, along with many other countries, has been actively exploring ways to improve access to HIV testing and treatment services for affected individuals and communities. However, in many settings, HIV patients are lost at each step of the continuum of HIV care [6–10]. Poor engagement in HIV care remains a major obstacle for the successful implementation of the Treatment as Prevention (TasP) strategy in China. Data from 2009 showed that only about half (53.3%) of HIV-positive individuals were tested for CD4 cell count within six months of the diagnosis of HIV infection [11]. Despite the provision of nation-wide HIV testing services and free ART programs, the proportion of ART-eligible HIV-positive patients who successfully receive treatment remains low (63.4%) [3].

In 2011, Guangxi Zhuang Autonomous Region (Guangxi) reported the highest number of AIDS-related deaths among all 31 provinces, autonomous regions and municipalities in China, accounting for 22% of the national total[12]. An earlier study suggested that in Guangxi, 50.4% of those who screened HIV-positive in hospital settings received a confirmatory Western Blot test, and 76.0% of those confirmed to be HIV-positive received their HIV test results. Of the individuals who were notified of their HIV-positive status, 31.4% did not receive CD4 testing [13]. Since CD4 cell count levels are used to determine ART eligibility (the criterion was CD4 ≤350 cells/mm3 or an AIDS diagnosis, at the time when the study was conducted), it was estimated that nearly 80% of newly identified, ART-eligible patients in Guangxi were not receiving ART timely [12].

Locally established practices, including the lengthy and complicated HIV referral process, were felt to be the primary drivers of low rates of confirmatory testing completion and ART initiation in this rural setting. In brief, the current standard of care following an initial positive HIV screening test calls for a subsequent repeat screening test, followed at a subsequent visit by an HIV confirmatory test (the Western Blot test), which is sent off-site and generally takes 10–15 days to complete. The patient is then expected to return to the facility for results and post-WB test counseling. At this visit, a blood sample is typically collected for baseline CD4 cell count measurement, which takes an additional 10 to 15 days. Patients must return to the facility or be located to learn their CD4 cell count test result and receive counseling regarding ART eligibility. This process inevitably delays ART initiation and results in a high proportion of EIA screened HIV positive patients being lost to follow up, thereby missing opportunities for patient engagement in HIV care, and posing significant barriers to achieving effective treatment outcomes for patients that ultimately translates into high mortality rates [14,15].

Although data are available on patients with a confirmed diagnosis of HIV infection, the current surveillance system does not actively monitor the testing completeness status of newly screened HIV cases prior to ART initiation (that is, the percentage of people who receive an HIV diagnosis after obtaining a positive HIV screening result). This prevents researchers and policy-makers from gaining an accurate understanding of how linkage to HIV treatment and care from testing can be further improved to enhance the continuum of care.

In order to develop strategies to streamline the approach and improve patient engagement in care in hospital based settings, a study was designed and implemented by the National Center for AIDS Control and Prevention (NCAIDS), Chinese Center for Disease Control and Prevention, to identify the limitations of the current routine testing practices along the HIV continuum of care. We report here on the results of the study in the 18 selected counties in Guangxi, China.

## Materials and Methods

### Study Design

The present study included:

1. A cross-sectional review of hospital records to assess the proportion of newly screened HIV cases that completed testing and ART initiation in each study hospital. For this purpose, hospital records in 18 county general hospitals for the period from January 1, 2013 to June 30 2013 were reviewed to identify the number of newly screened HIV positive cases, their testing completeness and ART initiation status. Original hospital screening records were crosschecked against the data collected and stored in the National Comprehensive Response Information Management System (CRIMS).
2. A status quo mapping of HIV testing to ART initiation processes, and deviations from the standard of care. Investigators observed and documented key characteristics of the routine care practice adopted at each county, which included: the types of test kits used at each hospital for HIV screening, number of HIV screening tests required for an individual to be considered as a screened positive, number of blood draws required to achieve testing completion, number of hospital visits needed to achieve testing completion, frequency of blood sample delivery for off-site confirmatory testing, and cost of baseline physical examinations. Variations from standard of care were documented and summarized. A detailed pathway flowchart from HIV screening to treatment was developed for each of the counties, documenting the patient referral process and key deviations from the national guidelines for HIV testing and care services.
3. A survey of hospital organizational and staffing characteristics. Hospital characteristics such as number of beds, annual number of inpatients/outpatients, number of clinicians for HIV care were collected using a separate survey for the purpose of baseline stratification for the CTN-0056Ot study.

### Study Site

State-owned hospitals in China are classified by a 3-tiered system based on the hospital’s capacity to provide medical care and medical education and to conduct research. Level 1 Hospitals are generally community-based or township hospitals with fewer than 100 beds and are mainly responsible for providing preventive care. Level 2 Hospitals have between 100 and 500 beds, and are located in counties or districts. These hospitals offer a range of comprehensive health services, and have the capacity to provide some medical education and conduct limited research at the regional level. Level 3 Hospitals are city, provincial or national-level hospitals that are typically responsible for the provision of specialized medical services, usually with a bed capacity exceeding 500. These hospitals offer a wider range of services, and have higher capacity to provide medical education and conduct research. Within this 3-tiered-system, hospitals are further rated by 3 grades (A, B and C) based on hospital size, level of medical technology, service provision and quality of service, resulting in a total of 9 levels. There is also one additional special category (AAA) for the highest-ranked medical facilities, also known as the Level 3 Triple A Hospitals [16].

Each county usually have one county general hospital, one county woman and child care hospital, and one county hospital of traditional Chinese medicine and many township hospitals. Policies and practice of recommending HIV screening test varies among hospitals. HIV screening test is recommended for all patients who are hospitalized. For out-patients, HIV screening test is recommended if a patient has infections, or the doctor believes it is needed. Usually, some 3% to 10% of out-patients are recommended for HIV screening test. For pregnant women attending antenatal care at the woman and child care hospital, everyone is recommended for HIV screening test.

In Guangxi, counties usually have one Level 2A general hospital, which is designated as the only medical facility for delivering ART services. Level 2A general hospitals were selected for this study because they are also the largest health care facilities at the county-level in China’s hospital system, with the greatest number of inpatients and outpatients. Almost one-third of the total number of HIV cases in China are identified and reported from these hospitals[12].

Selected study hospitals had to have reported at least 20 HIV-positive screening cases in the 6-month period from January 1, 2013 to June 30, 2013. It should be noted that it was impossible to determine the number of newly screened positive cases from the reported number of HIV-positive screening cases, as the current HIV surveillance does not distinguish between newly screened and previously screened positive cases. Geographical location was also taken into account to ensure coverage of the entire Guangxi region. The study team travelled to twenty-four hospitals that fulfilled the above requirements. Eighteen hospitals were selected for final statistical analysis, excluding hospitals with missing values and those with fewer than 5 newly screened HIV-positive cases.

### Study Participants

Study participants included locally newly screened HIV positive individuals who were at least 18 years old, seeking care in the selected hospitals between January 1 and June 30, 2013. Prisoners, detainees and pregnant women were excluded. This is because, in Guangxi, all prisoners or detainees who test HIV positive are followed up by the local Centers for Disease Control and Prevention as well as medical staff at the detention facilities under a separate system and do not receive HIV care at the general hospital. Pregnant women receive HIV care services at separate specialized maternity care hospitals.

### Data Collection

A standardized data collection form was developed a priori to accurately capture all the required information. All site investigators were trained prior to the investigation.

Investigators engaged with hospital leaders and key personnel involved in HIV prevention activities, which usually included one ART Clinician, one Hospital Laboratory Staff, one Hospital Case Report Officer and one to two members from the County CDC HIV Division.

Information collected in this study has been used as baseline data in the *CTN-0056Ot Testing and Linkage to HIV Care Clustered Randomized Controlled Trial Study (NCT02084316)*, co-funded by the U.S. National Institute on Drug Abuse (NIDA), and the China National Health and Family Planning Commission (NHFPC). This ongoing prospective study assesses the effects of a structural intervention to reduce the interval from HIV screening to ART initiation in rural China.

### Statistical Analysis

Statistical analyses presented are purely descriptive, where data within hospitals are tabulated and summarized across the 18 hospitals, using means, medians, 25^th^ and 75^th^ quartiles, and ranges. The total number of newly screened HIV-positive cases identified between January 1, 2013 and June 30, 2013, along with the numbers and proportions of newly screened cases that successfully received both the Western Blot and CD4 testing with counseling were tabulated and stratified by time-points, at 30 days, 60 days, 90 days, and up to the point of final observation. We documented the interval (days) from the initial positive EIA screening to confirmatory HIV testing (the Western Blot test, and the CD4 measurement) and counseling. The ART initiation success rates for ART-eligible patients (i.e.: CD4 count ≤ 350 cells/mm^3^) were tabulated by Study County. More detailed information about completion of WB test, CD4 test and initiation of ART in the 18 hospital are available in S1 Dataset.

### Ethics

This study was a secondary data analysis using existing hospital records. All patients signed an informed consent upon for taking HIV test saying that their data, after removing their personal identifications, could be used in epidemiological studies such as this. Therefore, no additional study-specific consent for this current study was sought. The survey was structured so that all personally identifiable information was excluded. The patient records was de-identified and anonymized prior to analysis. The study was reviewed and approved by the Institutional Review Board of the National Center for AIDS/STD Control and Prevention, Chinese Center for Disease Control and Prevention.

## Results

The 2009 National Guidelines for HIV/AIDS Detection recommend that when a patient is screened HIV positive on the initial screening, two subsequent repeat screening tests should be carried out at the same time (preferably using different testing methodologies or reagent brands from the initial screening)[17]. Only 5 hospitals out of the 18 hospitals followed the national guidelines in this respect. Three County CDCs conducted EIA tests regardless of patients’ screening results. The Guidelines [18] also recommend that a new blood draw always be carried out when a patient needs to be tested for HIV confirmation in order to reduce contamination rates and mishandling of blood samples. However, ten County CDCs used the same blood sample collected for EIA screening to carry out the WB confirmatory testing. Additionally, the Guidelines recommend that a patient should always return to the hospital to collect his or her test result and receive counseling. However, in most of the counties visited, after the initial EIA screening at the hospital, patients would have to make at least one and often two trips to the county CDC to collect their WB and CD4 cell count test results.

A total of 699 eligible patient records from 18 county hospitals were reviewed and included in the study, with an average of 39 newly screened HIV-positive patients per hospital. The lowest hospital identified 15 newly screened HIV-positive patients and the highest 73. Details on the number of newly screened HIV-positive cases and their testing completeness rates in 18 Level 2A county-level general hospitals across Guangxi from January 1 to June 30 in 2013 are shown in **Table 1**. The proportion of newly screened HIV positive individuals who successfully achieved testing completion (i.e. HIV screening, Western Blot test, CD4 cell count test) within 30 days of their HIV-screen positive result averaged across the 18 hospitals was 25.5% (range of 0 to 86.7%, median 15.3%). This average percent across the hospitals increases to 43% (13.6–86.7%, median 41.8%) for patients achieving testing completion at any time after receiving a positive screening result, and is 37% (2.3–86.7%, median 39.8%) within 60 days, and 41.8% (13.6–86.7%, median 41.8%) within 90 days. The proportion of patients who were confirmed to have HIV (positive Western Blot) among those that did have a Western Blot performed was 96.9%.

**Table 1:** The number of newly screened HIV positive cases and the testing completeness proportions in 18 county general hospitals in Guangxi between January 1 and June 30, 2013.

Hospital code	Testing Completeness Rates								
	Initial HIV EIA screen positive that are eligible for the study	Complete WB&CD4 in 30 days	Proportion (%) of WB & CD4 with counselling in 30 days	Complete WB & CD4 with counselling in 60 days	Proportion (%) of WB & CD4 with counselling in 60 days	Complete WB & CD4 with counselling in 90 days	Proportion (%) of WB & CD4 with counselling in 90 days	Complete WB & CD4 with counselling up to the point of observation	Proportion (%) of WB&CD4 with counselling up to the point of observation
1	44	0	0	1	2.3	6	13.6	6	13.6
2	29	0	0	4	13.8	5	17.2	5	17.2
3	40	2	5	6	15	7	17.5	7	17.5
4	33	2	6.1	14	42.4	16	48.5	16	48.5
5	41	4	9.8	8	19.5	10	24.4	14	34.1
6	36	4	11.1	7	19.4	8	22.2	8	22.2
7	34	4	11.8	10	29.4	14	41.2	14	41.2
8	40	5	12.5	8	20	10	25	11	27.5
9	29	4	13.8	7	24.1	10	34.5	10	34.5
10	48	8	16.7	21	43.8	24	50	26	54.2
11	43	13	30.2	16	37.2	16	37.2	16	37.2
12	47	15	31.9	21	44.7	24	51.1	24	51.1
13	26	10	38.5	11	42.3	11	42.3	11	42.3
14	33	13	39.4	15	45.5	17	51.5	17	51.5
15	73	32	43.8	38	52.1	41	56.2	41	56.2
16	37	18	48.6	24	64.9	24	64.9	25	67.6
17	51	27	52.9	32	62.7	35	68.6	36	70.6
18	15	13	86.7	13	86.7	13	86.7	13	86.7
Average	39	10	25.5	15	37.0	17	41.8	17	43.0
Median (25^th^,75^th^ quartile)	39 (33, 44)	7 (4, 13)	15.3 (10.1, 39.2)	12 (8, 20)	39.8 (34, 45.3)	14 (23, 33)	41.8 (33, 51.4)	14 (22; 32)	41.8 (31, 53.5)

We also calculated the average number of days within hospital from receipt of initial HIV positive EIA screening result to receipt of confirmatory results (the Western Blot test, and the CD4 cell count test) and completion of counseling (**Table 2**). The average time from HIV positive screening to receipt of confirmatory results and counseling across the 18 hospitals was 41.7 days (14–116 days, median 38 days), with a total of 11 out of 18 (61%) hospitals reporting an average interval longer than 30 days (range: 116 to 14 days). At the individual patient level, the shortest interval was 0 day and the longest 260 days.

**Table 2:** Average time interval from initial EIA HIV screening to confirmatory counselling in 18 county hospitals in Guangxi, China.

Hospital code	Average time interval between EIA positive and WB&CD4 counselling (days)	Range of time interval between EIA positive and WB&CD4 counselling (days)
1	14	1–30
2	19	11–32
3	22	11–54
4	27	6–91
5	28	15–79
6	28	9–126
7	29	3–83
8	33	16–62
9	38	25–65
10	38	0–260
11	41	20–78
12	41	20–105
13	44	19–111
14	50	34–73
15	54	14–208
16	59	14–179
17	69	13–167
18	116	49–183
Average	41.7	Maximum range of duration: 0–260
Median (25%,75% quantile)	38 (28, 49)	

The proportions of newly diagnosed HIV cases that initiated ART for each of the hospitals are presented in **Table 3**. The proportion of all HIV positive individuals that initiated treatment during observation averaged across the 18 hospitals was 21.8% (2.5–66.7%, median 19.9%). The hospital-averaged proportion of newly diagnosed HIV positive individuals that had initiated treatment within 90 days of the initial EIA screening regardless of their treatment eligibility status was 19.4% (2.5–66.7%, median 15.7%). The average proportion of ART-eligible (i.e.: CD4 count ≤350 cells/mm3) individuals that initiated treatment within 90 days was 18.0% (2.5%-66.7%, median 15.1%).

**Table 3:** The proportions initiating treatment in 18 county hospitals in Guangxi between January 1 and June 30, 2013.

Hospital code	Proportion of treatment (CD4≤350)			Proportion of treatment (all)		Proportion of EIA positive that initiated ART up to the point of observation	
	Initial HIV EIA screen positive	Initiated ART in 90 days	Proportion (%) of CD4≤350 that initiated ART within 90 days	Initiated ART in 90 days	Proportion (%)	Initiated ART up to the point of observation	Proportion(%) of EIA positive that initiated ART up to the point of observation
1	44	2	4.5	2	4.5	3	6.8
2	29	1	3.4	1	3.4	2	6.9
3	40	1	2.5	1	2.5	1	2.5
4	33	9	27.3	9	27.3	10	30.3
5	41	8	19.5	9	22.0	9	22.0
6	36	2	5.6	2	5.6	2	5.6
7	34	3	8.8	4	11.8	7	20.6
8	40	4	10.0	4	10.0	4	10.0
9	29	6	20.7	7	24.1	7	24.1
10	48	8	16.7	9	18.8	11	22.9
11	43	5	11.6	7	16.3	8	18.6
12	47	6	12.8	7	14.9	9	19.1
13	26	5	19.2	6	23.1	6	23.1
14	33	5	15.2	5	15.2	5	15.2
15	73	11	15.1	11	15.1	14	19.2
16	37	10	27.0	10	27.0	11	29.7
17	51	19	37.3	21	41.2	25	49.0
18	15	10	66.7	10	66.7	10	66.7
Average	39	6	18.0	7	19.4	8	21.8
Median (25^th^, 75^th^ quartile)	39 (33, 44)	6 (3, 9)	15.1 (9.1, 20.4)	7 (4, 9)	15.7(10.4, 23.9)	8 (4, 10)	19.9 (11.3, 23.9)

**Fig 1:**
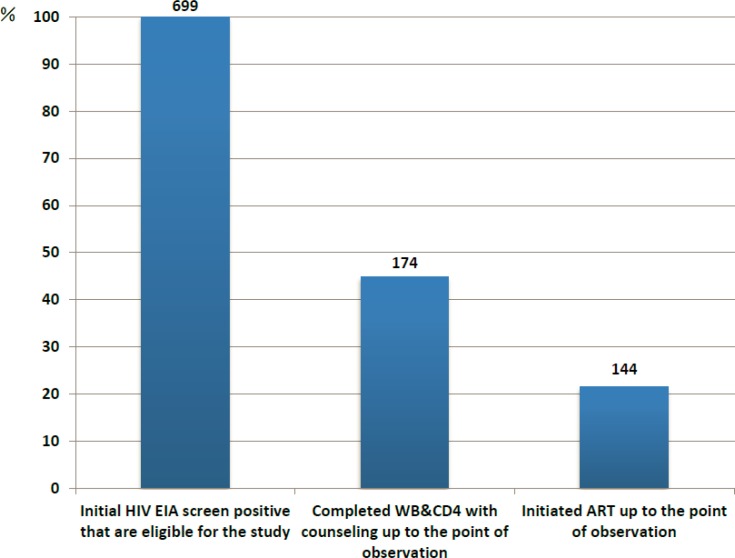
Cascade of Western Blot (WB) and CD4 Cell Count Testing Completion, Antiretroviral Therapy (ART) Initiation in 18 County Hospitals in Guangxi between Jan-Jun 2013.

HIV care cascades by testing completeness and ART initiation status are shown in **Fig 1**. Among all identified patients newly screened HIV-positive between January 1 and June 30, 2013, 55.2% of patients did not achieve testing completion. 78.5% of patients were lost between the initial HIV screening test and ART initiation. Only 17.2% of treatment-eligible patients successfully initiated ART treatment within 90 days after the initial HIV screening test.

## Discussion

Previous limited studies conducted in Guangxi suggested that only some 50% of patients screened HIV-positive received confirmation testing. Our study suggests that the figure could be even lower at the county level at 43%. Our results also confirm that only 22% of newly identified ART-eligible patients in Guangxi are engaged in HIV treatment timely, an alarmingly low treatment coverage rate compared to other low- and middle-income countries[19].

A major cause for the low testing completion and treatment initiation in rural Guangxi is the complicated and lengthy diagnostic pathway, and the disjointed HIV services, which are constrained by local structural and cultural factors. Currently, there are huge variations in the quality of HIV care offered in different counties. County hospitals and CDCs often struggle to follow national guidelines on standard of care for HIV services, mainly because of laboratory capacity limitations and the complexity of the referral system between hospitals and CDCs. When patients are screened HIV positive, they often need to travel between the county hospital and the county CDC several times in order to obtain confirmation of their HIV status, CD4 cell count testing, counseling, and subsequently initiate antiretroviral treatment.

Based on observations and conversations with the local staff members, we have learned that some patients refused to undergo further HIV confirmatory testing as a result of being afraid of related stigma and discrimination. This was consistent with our finding of a deep-rooted stigma against HIV patients among the leaders and staff of local hospitals and CDCs providing HIV care. Further, some officials and staff openly expressed their support for the use of punitive measures, such as public shaming or revealing HIV positive individuals’ identity, as effective ways to curb HIV rates among lower-income sex workers. In addition, we learned that at least some of the patients might have been turned away by hospital staff in an effort to avoid patient follow-up and reporting to the national database. Counseling before HIV screening test and personal contact information collection (address, contact number, etc.) after a positive screening result were also important reasons for lost to follow-up.

There are two major underlying causes of this huge variability of standards of care across hospitals. Firstly, most of the county-level hospitals follow a traditional stovepipe management structure, where very little cross-organizational communication is promoted. Staff involved in HIV testing and care (such as laboratory staff, Case Report Officers and ART clinicians) have little interest in following up with the patients to ensure that adequate care is provided. There are no assigned hospital staff to follow up on patients who do not return for their test results. In addition, CDCs and hospitals have been operating under two separate and distinct jurisdictions, each with its own independent funding source from different government agencies. The success of a patient receiving treatment through the current HIV testing process is largely reliant on the relationship between the hospital and CDC at the county level. Many patients are lost to follow up during this referral process, further highlighting the need to streamline the current standard of care practice and co-locate HIV care services within one setting.

We also found that there is limited hospital staff capacity, which conspires against active follow-up of patients during the pre-ART phase. More than half of the hospitals (13) reported that they are struggling to attract and retain health workers that are willing to work in HIV care because of stigma, misconceptions of occupational exposure and financial disincentives. Although public hospitals are by definition not-for-profit, all of them are under pressure to generate revenue to cover operational and administrative costs, and turn earnings into additional infrastructure whenever possible[20]. As most of the services related to HIV care are free of charge generating no revenue, hospital administrators have no incentive to increase their capacity in the provision of these services. At public hospitals, doctors and nurses are salaried employees. While salaries are based on work experience and professional level, some hospitals also pay a bonus to health workers according to their workload [21]. Given that HIV services are free of charge and cannot directly generate profit for the hospitals, doctors and nurses that work in HIV care often do not make as much money as their peers. While the hospital leaders acknowledge the need for reform, they have little interest in supporting initiatives to increase staff capacity or increase wages in the long term without additional financial support from the government.

Another challenge for patients to successfully access HIV care in the current hospital care setting is the high financial burden on HIV patients despite seemingly free HIV care. Across the 18 hospitals investigated, the typical cost of a baseline physical examination before ART treatment initiation ranged from 200-600RMB ($33–100 USD). While 90% of this is eligible for reimbursement, the upfront cost represents a substantial barrier to access, particularly among individuals with less economic resources or other competing needs. Only three counties at the time of this research had implemented a fee exemption policy. Hospital and CDC administrators also expressed concerns about patients’ financial hardship. In some counties, patients would also have to pay for medical fees incurred as a result of opportunistic infections.

There are a few limitations that should be noted in our calculations for testing completeness and treatment proportions. We have limited our study hospitals to only county general hospitals that had a minimum of 20 HIV+ patients during the study period. We also excluded prisoners, detainees and pregnant women from this study. Therefore, the results do not represent the full spectrum of the HIV care cascade. Eight site investigators conducted our investigation during different time periods. There may exist some degree of researcher subjectivity bias. Additionally, while we stressed that the investigation was not to evaluate the performance of each hospital, hospital staff may have misreported their current testing practices due to fear of being held responsible.

## Conclusions

In order for HIV-positive patients to fully benefit from ART, they need to know their HIV status, be productively engaged in HIV care, start ART in a timely fashion and adhere to their ART regimen on a long-term basis [5]. These represent the basic principles of the proposed United Nations 90-90-90 Target [22], which was endorsed by China within the BRICS declaration in late 2014 [23]. Our survey has shown that the current HIV testing practices and subsequent care procedures vary considerably across the county hospitals in Guangxi, which reaffirms the need for a simpler, streamlined service model to promote quicker, more efficient flow of patients from HIV screening to ART initiation. A critical paradigm shift needs to take place in the mode of service delivery at the county-level, where HIV affected individuals be able to gain access to the full spectrum of HIV care within one medical setting, and within an abbreviated timeframe. This could be greatly facilitated by the adoption of a seek, test, treat and retain model [24], which proposes universal ART treatment to all HIV infected individuals regardless of symptoms or CD4 cell count level, as it is currently increasingly recommended in some international guidelines [25,26]. These will be critical next steps if China is to optimally benefit from the promise of HIV Treatment as Prevention.

## Supporting Information

S1 Dataset (XLSX)
